# Postpartum family planning counselling during maternity care visits in Bangladesh and its effect on contraceptive initiation

**DOI:** 10.7189/jogh.14.04246

**Published:** 2024-12-20

**Authors:** M Moinuddin Haider, Md Mahabubur Rahman, Shusmita Khan, Tasnuva Khan Efa, Mizanur Rahman

**Affiliations:** 1 Health Systems and Population Studies Division, International Centre for Diarrhoeal Disease Research, Bangladesh (icddr,b), Dhaka, Bangladesh; 2 Data for Impact, University of North Carolina at Chapel Hill, Chapel Hill, USA; 3 Maternal and Child Health Division, icddr,b, Dhaka, Bangladesh

## Abstract

**Background:**

Postpartum family planning (PPFP) is an essential component of birth care that helps avert maternal and newborn health hazards by preventing short-spaced births. Many Asian and African studies found PPFP counselling during antenatal care (ANC) and postnatal care (PNC) effective in increasing PPFP uptake. Studies in Bangladesh, however, provided limited evidence of the feasibility and effectiveness of integrating PPFP in maternal health services. The national action plan integrated PPFP services (counseling and providing methods) in maternal health care and immunisation programmes. However, no study has examined the availability of PPFP counselling, an essential component of PPFP, in maternity care points and its effectiveness in increasing PPFP initiation. We explore the prevalence and correlates of PPFP counselling during ANC and PNC and investigate whether PPFP counselling during ANC and PNC increases PPFP initiation.

**Methods:**

We used nationally representative data from the 2017–18 Bangladesh Demographic and Health Survey to analyse whether women having the last live birth in the past three years received PPFP counselling during ANC or PNC visits. We included women’s other characteristics as covariates in a multivariable logistic regression. Finally, we analysed the 12-month PPFP initiation by PPFP counselling during ANC and PNC visits. The PPFP initiation analysis used self-reported contraceptive calendar data, a life table technique, and a proportional hazards model.

**Results:**

The prevalence of PPFP counselling was 12% during ANC and 22% during PNC. Women with higher education, higher birth order, upper household wealth quintiles, and living in the Khulna division compared to Chattogram (i.e. the division with the lowest PPFP counselling prevalence) were more likely to receive PPFP counselling during ANC and PNC. Three-fourths of the women initiated FP within 12 months postpartum. PPFP initiation was higher for women receiving PPFP counselling during PNC than those who did not receive it during PNC. We did not find evidence of increased PPFP initiation among women receiving PPFP counselling during ANC.

**Conclusions:**

The higher PPFP initiation among women receiving PPFP counselling during PNC is encouraging. Although we did not find evidence supporting increased PPFP initiation among women receiving PPFP counselling during ANC, further investigation on the quality of PPFP counselling during ANC may guide this necessary intervention’s implementation and scale-up.

The World Health Organization (WHO) defines postpartum family planning (PPFP) as ‘the prevention of unintended pregnancy and closely spaced pregnancies through the first 12 months following childbirth’ [1]. Thus, by definition, PPFP is not limited to the use of contraception; instead, it is a high-impact practice and an essential component of birth care. It prevents short-spaced birth (birth to next conception interval shorter than 24 months), which is a risk factor for adverse maternal and newborn health outcomes like maternal anaemia, puerperal endometritis, premature rupture of membranes, maternal mortality, premature birth, low birth weight, and infant mortality [1–4].

The global burden of short-spaced birth is heavy in low- and middle-income countries (LMICs), where around one-quarter of the second and higher-order births are short-spaced [1,5]. Bangladesh is the second among the 90 Demographic and Health Survey (DHS) countries in terms of its lowest proportion of women having short birth intervals [6]. Still, during 2016–18, 11% of women who had a birth in the past three years reported that their birth interval was less than 24 months and 15% of women reported a birth interval between 24–35 months [6]. Infant and under-five mortality was about two times higher among newborns who were born within 24 months of the birth interval than those who were born later, according to the 2017–18 Bangladesh DHS [6]. In addition to an estimated 0.2 million short-spaced births (calculated), approximately 1.6 million abortions and menstrual regulations also happen every year in the country, many of which are likely to be associated with short-spaced births [7].

Initiating a family planning (FP) method immediately after delivery, preferably on the delivery table, can be a straightforward and practical approach to prevent short spacing between births, particularly when continued until planning the next conception with appropriate birth spacing. However, even after resuming sexual activity, many women may not use any FP method until after experiencing their first postpartum menstruation. This delay often stems from a lack of awareness, particularly in developing countries, where women may not realise that postpartum menstruation is not necessarily anovulatory [8–11]. There is a common misconception that a woman cannot conceive until after her first postpartum menstruation. Menstruation is the end of a cycle which starts with ovulation and, after the end of a pregnancy, ovulation can occur as early as 28 days postpartum, which leaves 10–44% of women at risk of pregnancy during the postpartum period [12].

Despite the importance of PPFP in preventing short-spaced births and reducing its subsequent adverse consequences on maternal and newborn health, its acceptance rate is 74% in Bangladesh [13]. Although the rate is relatively high, the other 26% remain at risk of short-spaced pregnancies. The median duration of postpartum abstinence is only 2.8 months, indicating that more than half of the postpartum women in the country are at risk of pregnancies beginning in the third month postpartum and beyond, unless they use an FP method [6]. Many women may also plan the subsequent pregnancy in the postpartum period, meaning they possibly do not envision the health consequences of short birth spacing and, therefore, do not accept FP immediately after birth. Thus, there is full scope for further reducing short-spaced births by increasing PPFP use through effective intervention, which eventually helps reduce infant and under-five mortality, and abortion/menstrual regulations.

Many studies in different countries investigated and recommended potential ways to improve PPFP. We have cited a few as references in the previous discussion; however, many more such studies exist. The WHO and United States Agency for International Development’s (USAID) Knowledge SUCCESS programme precisely summarised the following possible intervention points to increase PPFP use [1,14]:

− PPFP counselling during facility-based ANC and community-based pregnancy screening (in cases when women do not go to facilities for ANC).
− PPFP counselling and appropriate services during any contact with women delivering in facilities or at home by skilled birth attendants during the delivery and 48-hour postpartum periods.
− PPFP counselling and services to women whenever they receive PNC – i.e. either at facilities or at home during the 48-hour to six-week postpartum period.
− PPFP counselling and services as part of the maternal and child health programme whenever the following occur (either at facilities or at home): immunisations, well-child visits, nutrition and growth monitoring, event days (such as during Vitamin A supplementation days), illness visits, and preventing mother-to-child transmission of HIV and other antiretroviral care during the period from six weeks to 12 months postpartum.

A review of studies from LMICs on the effect of counselling indicated that high-intensity FP counselling during ANC (e.g. multiple counselling sessions, counselling with a duration longer than that in the common practice of LMIC ANC providers) might help increase FP use [15]. Counselling of postpartum women before discharge from facilities is expected to impact subsequent FP use [15].

Many studies have investigated the needs, barriers, and enablers of PPFP among Bangladeshi women and recommended the implementation of PPFP counselling [16–18]. A sub-national level intervention study conducted during 2007–2009 provided evidence of the feasibility and effectiveness of integrating PPFP services in community-based maternal health services and services in Union Health and Family Welfare Centres [19]. Most women in recent years have received ANC (a large portion from health facilities), half of the births happen in health facilities, and more than half of the women receive at least one PNC within two days of birth [20]. That means women are in contact with medical care providers during the maternity period for maternal health care. Therefore, it is also important to understand the feasibility and effectiveness of PPFP counselling in facility-based maternity care points (ANC, delivery, and PNC). A recent tertiary-level health facility-based study aimed to improve postpartum intrauterine device (PPIUD) insertion through PPIUD training of the providers [21]. The study improved counselling, but not PPIUD insertion or acceptance. The evidence of improved counselling is encouraging. The context of historically low acceptance of IUDs in Bangladesh might have influenced the failure of the intervention to increase PPIUD insertion, which may not be the case for other contraceptives [6].

The National Technical Committee of the Directorate General of Family Planning (DGFP) in 2016 identified no or limited information about PPFP counselling during ANC, PNC, and child immunisation visits at any Expanded Program on Immunisation (EPI) sites [22]. Afterward, the National Technical Committee approved integrating information about FP during these health care points and the provision of providing short-acting methods (i.e. pills, condoms, and injectables) from the EPI sites [23,24]. The National Technical Committee laid out an implementation plan to generate demand through behaviour change communication, training of providers, availability of the FP methods, logistics and supply management, and quality improvement activities that are likely to increase PPFP use [22].

The above brief context analysis shows that many explorative studies suggested PPFP counselling, small intervention studies provided evidence of the feasibility of PPFP counselling at maternity care points, and the national action plans have approved PPFP services at maternity care points and EPI sites. However, no national-level study examined whether Bangladeshi women receive PPFP counselling, which is an essential component of PPFP services at maternity care points (ANC, delivery, and PNC visits), and whether the counselling improves PPFP acceptance among Bangladeshi women.

## Study objectives

The objectives of this study are two-fold: to examine the extent to which PPFP counselling are received by the women at maternity care points (i.e. during ANC and PNC visits) in Bangladesh and factors associated with the counselling; and to investigate whether the PPFP initiation among women who received PPFP and counselling during ANC and PNC visits was higher than those who did not.

## METHODS

We analysed secondary data from the 2017–18 Bangladesh DHS, a nationally representative cross-sectional survey, which adapted a two-stage stratified cluster sampling technique. The first stage involved selecting 620 enumeration areas (i.e. clusters) from rural and urban areas from eight divisions each. Then, in the second stage, 30 households from each cluster were drawn using systematic random sampling. All the ever-married women ages 15–49 living in the households were selected to be interviewed using a structured questionnaire. The survey eventually interviewed 19 445 households with a response rate of 99.4% and 20 127 ever-married women of reproductive age living in households with a 98.8% response rate.

The use of PPFP is the primary outcome of this study. Therefore, we included currently married women of reproductive age (CMWRA) because the socio-religious norms of Bangladesh do not allow and accept sexual union beyond wedlock. The analytical sample included 4842 CMWRA who had a live birth in the past three years before the survey and had passed at least one-month postpartum period.

### Construction of the dependent variables

#### Receiving PPFP counselling during ANC

The 2017–18 Bangladesh DHS asked women the question, ‘As part of your ANC during this pregnancy, did you receive counselling about a family planning method you can use immediately after you give birth?’, with yes/no being the available answers. Based on the question, we constructed the binary dependent variable, ‘Receiving PPFP counselling during ANC’ with yes and no categories, where ‘PPFP counselling’ refers to ‘counselling about the FP method use immediately after birth’.

#### Receiving PPFP counselling during PNC

The 2017–18 BDHS asked questions on PPFP counselling during PNC only to women who had facility birth and received PNC for their most recent live birth in the three years preceding the survey. Thus, analysis of PPFP counselling during PNC confines to women who had facility birth and received PNC within 42 days postpartum. We constructed the variable ‘Receiving PPFP counselling during PNC’ with two possible outcomes: received PPFP counselling and did not receive PPFP counselling.

#### 12-month PPFP initiation

The 2017–18 Bangladesh DHS collected data about the last FP use and adapted the contraceptive calendar method to collect month-wise FP use data over the previous five years. The contraceptive calendar method collects FP use, pregnancy, birth, and postpartum amenorrhea information for every month in the past five years using a systematic approach [25]. The primary outcome of this study is PPFP initiation. Therefore, we used the FP data collected using the contraceptive calendar method. We set a one-year postpartum period to create the follow-up time and censoring indicator for PPFP initiation. Event cases include CMWRA who initiated PPFP within the 12-month postpartum period. We classified the censored cases into two groups. The first censored group included CMWRA who completed 12 months postpartum but did not initiate PPFP, and the second group included CMWRA who neither completed the 12-month postpartum period until the survey visit nor initiated PPFP. For event cases that initiated PPFP, we constructed the follow-up time by subtracting the century month code (CMC) of the last birth from the CMC of PPFP initiation. For the first censoring group, the follow-up time was eleven complete months. We constructed the follow-up time for the second group by subtracting the CMC of the last birth from the CMC of the survey month.

#### Explanatory variables and reasons for inclusion of the variables

Explanatory variables include menstruation resumption after birth, the difference between total living children and the desired number of children, place of delivery, mode of delivery, and wantedness of the last child. Regarding background characteristics, we captured the CMWRA’s age at last birth, parity, religion, education, household wealth quintiles, urban/rural residence, and region. PPFP counselling during ANC and PNC are dependent variables under the examination of our first objective, but served as the explanatory variables in the analysis for our second objective. We have provided the variables with explanations and variable-specific categories with definitions in Appendix S1 in the **Online Supplementary Document**.

The conceptual understanding of the PPFP use and prior studies on this suggests that the previously discussed characteristics (the explanatory variables) can potentially influence a person's FP behaviour (e.g. care seeking, care receiving, method use) [26–29]. Therefore, they are included in obtaining adjusted results on PPFP counselling during ANC and PNC, as well as PPFP initiation.

### Statistical analysis

#### Sample characteristics

We used frequency and percentage distribution to understand the socio-demographic and other characteristics of the sampled CMWRA. The explorative analysis provides unweighted numbers, weighted numbers, and weighted percentages.

#### Analysis for first objective

We estimated the percentage of CMWRA who received PPFP counselling during ANC and PNC visits and whether receiving the PPFP counselling was associated with several factors, including the woman’s age, parity, religion, education, household wealth status, urban/rural residence, region, and type of ANC provider. A multiple logistic regression model explored the factors associated with PPFP counselling during ANC and PNC visits. To examine the factors associated with PPFP counselling during PNC, we have included two more factors – whether the woman received PPFP counselling during the ANC visit and the mode of delivery. Although the type of PNC provider is also expected to influence PPFP counselling, we have not conducted this analysis because PPFP counselling during PNC was only asked for facility birth; thus, all PNC providers were medically trained professionals.

#### Analysis for second objective

We constructed single decrement life tables of PPFP initiation by the status of receiving PPFP counselling during ANC and PNC. The life tables showed the percentage of CMWRA who initiated the FP method within 12 months postpartum. We used a Cox proportional hazard regression to investigate the association of PPFP counselling during ANC and PNC with PPFP initiation after controlling for factors likely to influence PPFP initiation, and an additional model with interaction between PPFP counselling during ANC and PNC to examine whether PPFP counselling at ANC modified the association between PPFP counselling at PNC and the PPFP initiation.

The regression models controlled for provider and CMWRA characteristics included in the analysis under the first objective, as well as menstrual resumption, place of delivery, the number of living children the woman had, and her desired number of children.

## RESULTS

### Sample characteristics

A majority of CMWRA (85%) were below 30 years of age at the time of the last birth; the child was the first or second order birth for more than 70% of the CMWRA; 66% had some secondary education (i.e. ever attended class 6 or any higher grade); 8% reported the last child was unintended; more than half had their menstruation return within three months of delivery; and more than half had the same or higher number of living children they desired (**Table 1**). Nearly nine in ten CMWRA received at least one ANC from a medically trained provider; half had facility delivery; one-third went through a caesarean section delivery; and 57% received at least one PNC from a medically trained provider.

**Table 1:** Characteristics of participants

Variables by category	Unweighted number (%) (n = 4779)	Weighted number (%) (n = 4824)
Age at last childbirth in years		
*<20*	1351 (28.2)	1408 (29.1)
*20–24*	1584 (33.0)	1560 (32.2)
*25–29*	1141 (23.8)	1154 (23.8)
*>30*	723 (15.1)	720 (14.9)
Parity		
*1–2*	3399 (70.8)	3438 (71.0)
*≥3*	1400 (29.2)	1404 (29.0)
Years of schooling		
*≤5*	1630 (34.0)	1641 (33.9)
*6–9*	2041 (42.5)	2107 (43.5)
*>10*	1128 (23.5)	1094 (22.6)
Religious affiliation		
*Islam*	4390 (91.5)	4444 (91.8)
*Other (mainly Hinduism)*	409 (8.5)	398 (8.2)
Wantedness of last child		
*Wanted then or later*	4407 (91.8)	4452 (91.9)
*Wanted no more*	392 (8.2)	390 (8.1)
TLC vs DNC		
*TLC<DNC*	2185 (45.5)	2218 (45.8)
*TLC = DNC*	1617 (33.7)	1628 (33.6)
*TLC>DNC*	984 (20.5)	984 (20.3)
*Other*	13 (0.3)	13 (0.3)
Menstruation resumption		
*Did not return*	1140 (23.8)	1134 (23.4)
*≤3 months of last birth*	2660 (55.4)	2659 (54.9)
*4–7 months of last birth*	640 (13.3)	675 (13.9)
*8–11 months of last birth*	359 (7.5)	373 (7.7)
Received at least one ANC from a MTP		
*Yes*	3931 (89.2)	3969 (89.2)
*No*	478 (10.8)	483 (10.8)
Place of delivery		
*Home*	2364 (49.3)	2401 (49.6)
*Facility*	2435 (50.7)	2440 (50.4)
Mode of delivery		
*Normal*	3187 (66.5)	3222 (66.6)
*Caesarean section*	1607 (33.5)	1613 (33.4)
Received at least one PNC from a MTP		
*Yes*	2565 (57.6)	2554 (57.4)
*No*	1885 (42.4)	1893 (42.6)
Household wealth quintiles		
*Lowest*	1030 (21.5)	996 (20.6)
*Second*	979 (20.4)	996 (20.6)
*Middle*	853 (17.8)	911 (18.8)
*Fourth*	955 (19.9)	988 (20.4)
*Highest*	982 (20.5)	951 (19.6)
Residence type		
*Rural*	3151 (65.7)	3544 (73.2)
*Urban*	1648 (34.3)	1298 (26.8)
Administrative division		
*Barishal*	508 (10.6)	273 (5.6)
*Chattogram*	800 (16.7)	1027 (21.2)
*Dhaka*	715 (14.9)	1246 (25.7)
*Khulna*	500 (10.4)	444 (9.2)
*Mymensingh*	582 (12.1)	415 (8.6)
*Rajshahi*	502 (10.5)	560 (11.6)
*Rangpur*	531 (11.1)	509 (10.5)
*Sylhet*	661 (13.8)	368 (7.6)

mo – months, MTP – medically trained provider, TLC – total living children, DNC – desired number of children, ANC – antenatal care, PNC – postnatal care

### Prevalence of PPFP counselling at maternity care points and associated factors

Only 13% of the CMWRA who received at least one ANC received PPFP counselling (**Table 2**). The level of PPFP counselling varied by different characteristics of the CMWRA; between 19% and 10%. The multiple logistic regression showed that CMWRA of three or higher parity, those who completed at least ten years of schooling, those from the highest household wealth quintiles, and those living in Khulna and Rangpur rather than those in Chattogram (i.e. division with the lowest PPFP counselling at ANC) were more likely to receive PPFP counselling during ANC visits (statistically significant with *P*-values ≤0.05).

**Table 2:** PPFP counselling at ANC and at PNC*

Variables by category	PPFP counselling during ANC			PPFP counselling during PNC		
% received*	**aOR (95% CI)**	***P*-value**	**% received†**	**aOR (95% CI)**	***P*-value**
Total	12.5			21.6		
ANC provider						
*Qualified*	12.7	ref				
*Unqualified*	10.2	0.95 (0.66–1.36)	0.762			
Received PPFP counselling at ANC					.	
*Did not receive*				17.1	ref	
*Received*				46.9	4.24 (3.17–5.67)	<0.001
*No ANC*				8.2	0.40 (0.11–1.44)	0.159
Mode of delivery						
*Normal*				19.9	ref	
*C-section*				22.4	1.18 (0.90–1.56)	0.239
Parity						
*1–2*	12.4	ref		20.3	ref	
*≥3*	12.8	1.34 (1.04–1.71)	0.021	26.7	1.39 (1.06–1.83)	0.018
Years of schooling						
*≤5*	9.5	ref		25.4	ref	
*6–9*	11.0	1.16 (0.88–1.53)	0.296	18.4	0.67 (0.48–0.95)	0.023
≥*10*	19.1	2.06 (1.49–2.85)	<0.001	23.4	0.75 (0.50–1.14)	0.182
Household wealth quintiles						
*Lowest*	10.0	ref		22.5	ref	
*Second*	10.0	1.02 (0.72–1.45)	0.894	18.4	0.85 (0.51–1.41)	0.530
*Middle*	11.2	1.14 (0.79–1.66)	0.474	17.8	0.80 (0.49–1.31)	0.374
*Fourth*	12.9	1.26 (0.88–1.80)	0.215	21.1	1.00 (0.63–1.60)	0.996
*Highest*	17.7	1.58 (1.05–2.36)	0.027	25.4	1.15 (0.69–1.91)	0.597
Religious affiliation						
*Islam*	12.3	ref		21.9	ref	
*Other*	14.2	1.07 (0.78–1.47)	0.678	18.5	0.77 (0.54–1.11)	0.158
Administrative division						
*Chattogram*	09.9	ref		20.7	ref	
*Barishal*	12.7	1.54 (1.00–2.38)	0.050	18.9	1.52 (0.95–2.43)	0.737
*Dhaka*	13.3	1.34 (0.91–1.96)	0.139	24.2	1.17 (0.81–1.69)	0.807
*Khulna*	13.0	1.51 (1.01–2.24)	0.043	14.7	0.87 (0.58–1.30)	0.119
*Mymensingh*	10.3	1.25 (0.83–1.88)	0.287	19.2	1.11 (0.73–1.69)	0.776
*Rajshahi*	12.4	1.53 (0.98–2.39)	0.063	15.1	0.77 (0.51–1.15)	0.131
*Rangpur*	17.7	2.42 (1.65–3.55)	<0.001	31.0	1.56 (1.01–2.41)	0.066
*Sylhet*	11.0	1.36 (0.84–2.21)	0.207	26.3	1.48 (0.96–2.28)	0.260
Residence type						
*Rural*	11.3	ref		20.1	ref	
*Urban*	15.4	1.19 (0.93–1.52)	0.172	24.5	1.02 (0.76–1.38)	0.896
Number of observations		4409			2388	

ANC – antenatal care, aOR – adjusted odds ratio, PNC – postnatal care, PPFP – postpartum family planning, ref – reference

*Denominator for PPFP counseling during ANC: who received ANC.

†Denominator for PPFP counseling during PNC: who had facility birth and PNC at facility.

Receiving PPFP counselling during a PNC visit was higher (22%) than receiving it during an ANC visit (13%). It was highest among CMWRA who received PPFP counselling during ANC (47%). The multiple logistic regression shows that CMWRA who received PPFP counselling during ANC, those with three or higher parity, and those with 6–9 years of schooling had a higher likelihood of receiving PPFP counselling during PNC visits (**Table 2**).

### PPFP initiation by PPFP counselling during ANC and PNC: descriptive and multivariate findings

**Figure 1:**
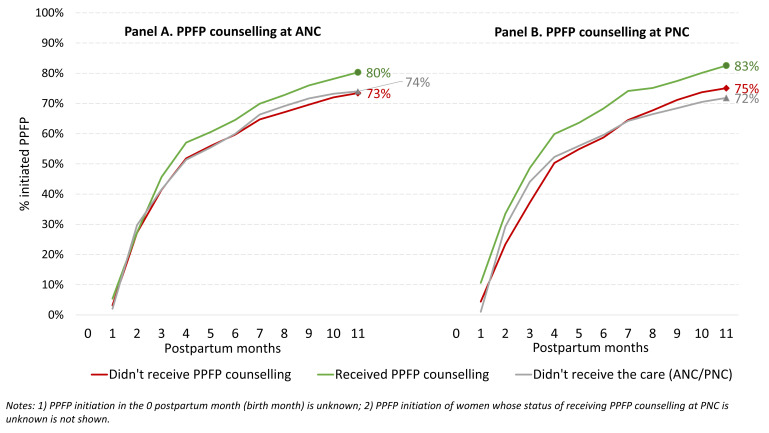
Postpartum months of initiating FP by PPFP counselling during ANC and PNC. **Panel A**. Twelve-month PPFP initiation rates by receiving PPFP counselling during ANC. **Panel B**. Twelve-month PPFP initiation rates by receiving PPFP counselling during PNC. ANC – antenatal care, FP – family planning, PNC – postnatal care, PPFP – postpartum family planning

CMWRA who did not go for ANC and those who did not receive PPFP counselling during ANC had the same probability of PPFP initiation (74%) (**Figure 1**, Panel A). Compared to them, PPFP initiation was higher (80%) among CMWRA who received PPFP counselling during ANC. The difference in PPFP initiation mainly appeared from the fourth month of postpartum and continued throughout the 12-month postpartum period. A higher probability of PPFP initiation appeared from the very first month of the postpartum period among CMWRA who received PPFP counselling during PNC compared to those who did not receive PPFP counselling during PNC or who did not receive PNC (**Figure 1**, Panel B). Among CMWRA who received PPFP counselling at PNC, 83% initiated PPFP within the first year of birth. Compared to them, PPFP initiation was lower among CMWRA who did not receive PPFP counselling during PNC (75%) and those who did not go for PNC (72%).

**Figure 2:**
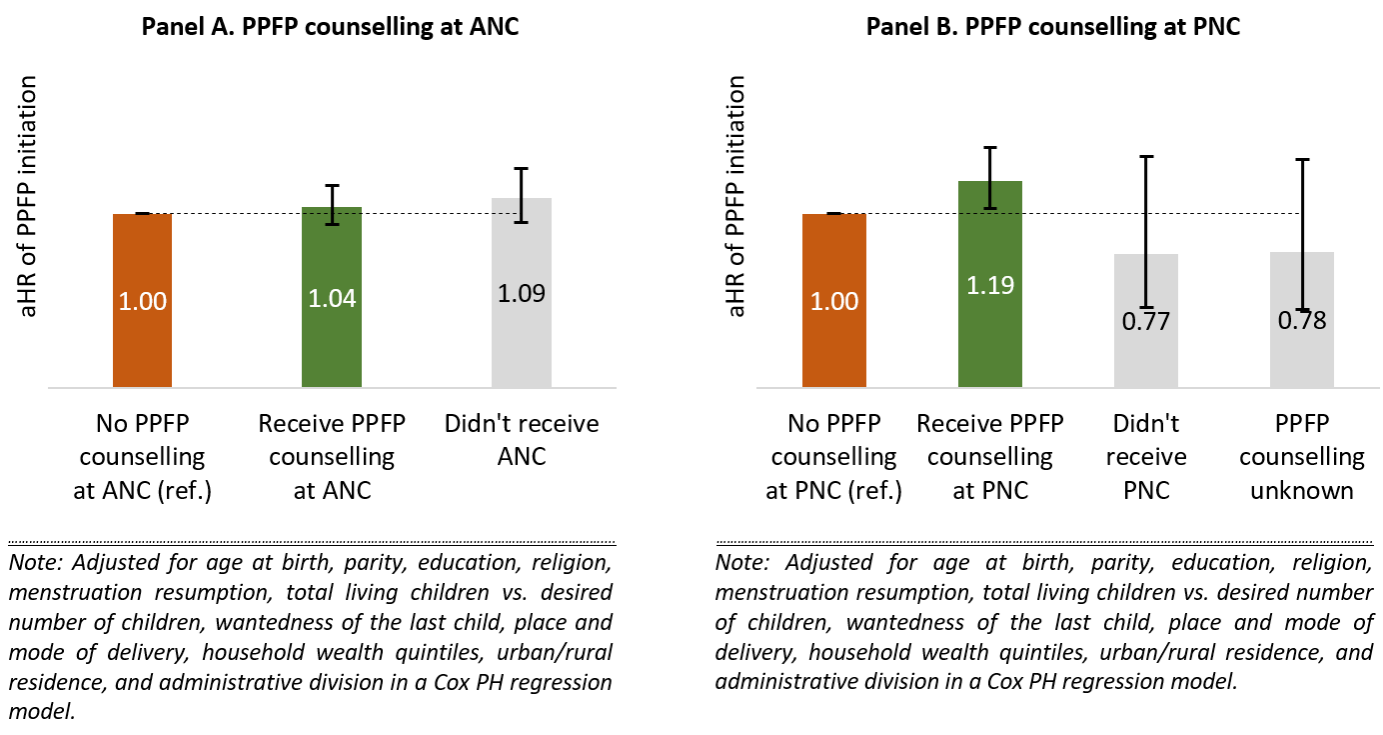
Adjusted hazard ratios (aHR) of initiating FP within 12 months postpartum by PPFP counselling during ANC and PNC. ANC – antenatal care, FP – family planning, PNC – postnatal care, PPFP – postpartum family planning

The hazard of PPFP initiation did not vary by PPFP counselling during ANC (**Figure 2**, Panel A; Appendix S2, Model 1 in the **Online Supplementary Document**). However, PPFP counselling during PNC was significantly associated with a higher hazard of PPFP initiation (**Figure 2**, Panel B). Compared to CMWRA who did not receive PPFP counselling during PNC, the hazard of PPFP initiation was 1.19 times higher among those who received PPFP counselling during PNC. Other correlates of PPFP initiation were menstruation resumption after birth, having the same or higher number of living children compared to the desired number of children, unwantedness of the last child, urban residence, and administrative division (Appendix S2, Model 1 in the **Online Supplementary Document**). Receiving PPFP counselling during both ANC and PNC did not make a difference in initiating FP within 12 months postpartum (Appendix S2, Model 2 in the **Online Supplementary Document**). The model found CMWRA who did not receive any ANC and whose receiving PPFP counselling at PNC status is unknown were more likely to initiate FP within 12 months postpartum. However, this is a very small group and therefore the finding should be used carefully.

## DISCUSSION

We examined the prevalence of PPFP counselling in Bangladesh during ANC and PNC and the factors influencing the counselling. Second, we investigated whether PPFP counselling during ANC and PNC improved FP initiation within 12 months postpartum.

We found a low level of PPFP counselling during ANC (13%) and PNC (22%). To understand this finding, we first need to explain the country context and whether there are FP activities that encompass PPFP services and counselling.

The FP programme in Bangladesh always embedded PPFP services. The government guidelines about PPFP service provision (i.e. providing counselling and methods) highlight ANC, delivery, PNC, and EPI contact points as PPFP service points [24]. Development partners (e.g. USAID and United Nations Population Fund) also work with government programmes to strengthen the PPFP services. For example, the Maternal and Newborn Care Strengthening Project funded by USAID worked with the government to improve PPFP counselling and service in 17 out of 64 districts, mainly in the central and eastern regions of the country [30]. It also implies that the PPFP counselling during ANC and PNC remains low, despite governmental and non-governmental efforts and investments.

The attitude and perception of maternal health care providers toward PPFP counselling during ANC and PNC may also be discussed to explain the low level of PPFP counselling during ANC and PNC. A widely-believed anecdotal thought is that many maternal health care providers do not perceive FP as part of women’s comprehensive health care; instead, they perhaps consider it to be just contraception and which FP method to use, and consider the PPFP counselling to be performed by FP providers [31]. There may be a lack of understanding among providers of the fact that birth spacing is a preventive measure against adverse infant and maternal health outcomes. The provision of health services under the Directorate General of Health Services (DGHS) and family planning services under the DGFP perhaps worsened the FP services at DGHS facilities and among DGHS providers. For example, providers in the Upazila Health Complex (a facility that accommodates both DGHS and DGFP providers) think the FP services should be provided by the FP providers in the same complex [31]. However, receiving PPFP counselling from DGFP facilities or providers at maternity care points is also low [32], implying a lack of understanding that PPFP use is not simply an FP issue, but also a health issue, and that maternal health care providers should be trained on this for practising PPFP counselling. Such training programmes about the benefits of PPFP must include providers from private facilities because they share a major stake in maternity care in Bangladesh.

Many providers also believe that ANC is not the proper time to provide PPFP counselling because the women are concerned about safe pregnancy and childbirth [31]. A recent study also showed the tendency of providers to offer PPFP counselling to higher parity (i.e. three or above) women [31]. It also limits the PPFP counselling to a small portion of women because three or higher parity women account for less than one-third of the recently delivered women. Additionally, a lack of adherence to working hours induces further challenges. A recent study in the Chattogram division found maternal health care providers in public health facilities available from 10 am to 1 pm instead of 9 am to 2 pm [31]. Such practice is also likely to be the case in other parts of the country. It increases the client load per hour and reduces the time needed for PPFP counselling. Private health care facilities and providers who provide ANC, delivery, and PNC services to more than half of the women are barely interested in FP services [20]. For example, the 2017 Bangladesh Health Facility Survey (BHFS) found only 53% of private health facilities offer any modern FP services, 25% provide any FP methods, and less than 5% have FP guidelines [33]. None of the private facilities in the 2017 BHFS sample were ready to provide short-acting FP services (none had all six items/components: FP guidelines, trained provider, blood pressure apparatus, pill, condom, and injectable) [33].

Lastly, the lack of coordination between DGFP and DGHS may be another source of low PPFP counselling during ANC and PNC. More than 90% of the union and above-level DGHS facilities reported to the 2017 BHFS that they provided PPFP services [33]. Although most women seek ANC and PNC services from DGHS facilities, we found only a few receive PPFP counselling at maternity care points which questions the FP service ownership by the DGHS facilities.

PPFP counselling during ANC and PNC is low, but a higher FP initiation within 12 months postpartum among those who received PPFP counselling during PNC is encouraging. It shows a potential intervention point to improve PPFP uptake by ensuring universal PPFP counselling coverage during PNC. The re-emphasising of the 2016 government circular on PPFP care at ANC, PNC, and EPI centres may play an important role in ensuring PPFP counselling during PNC [24].

Conversely, receiving PPFP counselling during ANC did not help improve PPFP initiation within 12 months postpartum. A recent Bangladeshi study also had similar findings [31]. However, many studies conducted in African and Asian countries found PPFP counselling during ANC a powerful intervention to improve PPFP uptake [15]. One of the reasons may be the high level of PPFP initiation in Bangladesh (74%). Increasing PPFP initiation from such a high level with any intervention is more difficult than increasing it from a low level. For example, Tafere et al. [34] showed PPFP counselling during ANC effective in increasing PPFP initiation in Northwest Ethiopia where the prevalence of PPFP was low (39% among those who received the counselling, and 19% among those who did not receive the counselling).

The unavailability of the quality of the counselling data (e.g. providing comprehensive FP information, sufficient time with the provider, and information about the importance of birth spacing, sharing or showing education and counselling materials, ensuring that the client understood the messages, etc.) weakens the findings. A recent study in Nepal found the lack of quality components of FP counselling during ANC (i.e. counselling for PPFP) as a major source of clients’ dissatisfaction with the counselling, which could also affect PPFP practice [35].

Moreover, many providers and clients do not prefer the ANC for PPFP counselling, as found in a recent study in Bangladesh [31]. Therefore, before concluding the association of PPFP counselling during ANC and its uptake within 12 months postpartum, further studies are needed to sensitise ANC providers about PPFP counselling. Proactive counselling may also help the client listen and adhere to the practice.

### The post-2017–18 government actions to improve PPFP services

This study uses data from the 2017–18 Bangladesh DHS. Therefore, the effect of any actions taken after 2017–18 has not been reflected in our findings. Before the 2017–18 Bangladesh DHS, the Government of Bangladesh developed a PPFP action plan in 2016, highlighting PPFP counselling as an essential action point. Based on the action plan, the DGFP also circulated a notice to provide PPFP services at ANC, PNC, and EPI centres and circulated two memoranda in 2019 about the provision of FP services in private hospitals and the provision of PPFP in public medical college hospitals, district, and upazila level public hospitals, as well as specialised public hospitals operated under the DGHS [36,37]. In addition to instructing the DGHS facilities to promote PPFP services, the circular informed that the wage compensation for service providers of long-acting reversible contraceptives and permanent methods and the clients would be disbursed through the DGHS system, which was not the case before 2019 [37]. Studies after 2019 may examine whether the 2019 circulars on PPFP made any changes.

### Strengths and limitations

This study is the first to examine whether PPFP counselling during ANC and PNC helps FP initiation within 12 months postpartum using a nationally representative survey (the 2017–18 Bangladesh DHS). Although it was a cross-sectional survey, the self-reported contraceptive history (calendar) of the 2017–18 Bangladesh DHS facilitated the longitudinal analysis, and incorporating postpartum month of FP initiation and menstruation resumption. The contraceptive calendar also allowed to include women who did not complete 12 months postpartum as censored cases.

However, the study also has a few significant limitations. The 2017–18 Bangladesh DHS did not collect data on whether the woman received PPFP information and counselling from other sources or points, what information was provided at the counselling, and the overall quality of the counselling. The lack of quality of PPFP counselling data limited the scope of examining the true effect of PPFP counselling on contraceptive initiation. Another limitation is the unavailability of information about PPFP counselling during delivery. However, a recent study shows that PPFP counselling is generally unavailable during delivery [18], so this likely did not affect our findings significantly. Researchers often interpret not perceiving FP as integral to health by DGHS providers as a potential barrier to FP services; however, scientific evidence supporting this is scarce. Rahman et al. [31] provided qualitative evidence in a recent study, but it was from a study conducted in only two districts.

Lastly, we do not know whether the provider initiated the counselling or the client asked for PPFP-related information. The conversation initiated by the client means that she had a demand for PPFP, so had a high likelihood of adopting it. Conversely, a conversation initiated by the provider might have provider bias in selecting a client to provide the counselling (e.g. women with higher parity). The availability of data on who exaclty initiated the conversation would provide the opportunity to examine whether there was any such selection introduced.

## CONCLUSION

PPFP counselling during ANC and PNC is low in Bangladesh. Moreover, PPFP counselling is associated with increased initiation of FP within 12 months postpartum during PNC, but not during ANC. However, several other studies in Asia and Africa found evidence of higher initiation of PPFP among women who received PPFP counselling during ANC. Therefore, we do not conclude that FP counselling during ANC does not help increase PPFP uptake. The lack of association may be due to the study’s limitations we reported. We suggest further studies to better understand the association of FP counselling during ANC and PPFP uptake in Bangladesh.

## Additional material


Online Supplementary Document
