# Supplementary material for: Postpartum family planning counselling during maternity care visits in Bangladesh and its effect on contraceptive initiation
Source: J Glob Health. 2024 Dec 20;14:04246. doi: 10.7189/jogh.14.04246 (PMC11658713; doi:10.7189/jogh.14.04246)
Supplement: Online Supplementary Document [file jogh-14-04246-s001.pdf]

2024 ● Vol. 14 ● 04246 doi: 10.7189/jogh.14.04246

Postpartum family planning counselling during maternity care visits in Bangladesh, and its effect on contraceptive initiation

M Moinuddin Haider,[1] Md Mahabubur Rahman,[1] Shusmita Khan,[2] Efa Khan,[3] Mizanur Rahman,[2]

[1]Health Systems and Population Studies Division, International Centre for Diarrhoeal Disease Research, Bangladesh (icddr,b), Dhaka, Bangladesh

[2]Data for Impact, University of North Carolina at Chapel Hill, Chapel Hill, USA

[3]Maternal and Child Health Division, icddr,b, Dhaka, Bangladesh

Appendix S1. Co-variates with the defined categories

Co-variates with categories	Description of categories of the co-variates
PPFP counselling during ANC	
Did not receive	Did not receive PPFP counselling at any ANC visits during the last live birth pregnancy
Received	Received PPFP counselling at any ANC visits during the last live birth pregnancy
No ANC	Did not receive ANC during the last live birth pregnancy
PPFP counselling during PNC	
Did not receive	Did not receive PPFP counselling during PNC
Received	Received PPFP counselling during PNC
No PNC	Did not receive PNC within 42 days of birth
PPFP counselling unknown	Received PNC within 42 days, but PPFP counselling information is unknown.
PNC receiving status unknown	Did not report about receiving PNC (excluded from analysis)
Menstruation resumption	
Did not return	Was amennorheic on the survey month
Within 3 months of last birth	Menstruation resumed within 3 months of the last birth
Within 4-7 months of last birth	Menstruation resumed between 4-7 months after the last birth
Within 8-11 months of last birth	Menstruation resumed between 8-11 months after the last birth
Total living children (TLC) and desired number of children (DNC)	
TLC < DNC	Had fewer living children than her desired number of children
TLC = DNC	Had as many living children as she desired
TLC > DNC	Had living children more than her desired number of children
Place of delivery	
Home	Delivered outside health facility
Facility	Delivered at a health facility
Mode of delivery	
NVD	Normal vaginal delivery
C-section	Caesarean delivery
Wantedness of the last child	
Wanted then or later	Wanted the birth at that time, or wanted it later but conceived early
Wanted no more	Did not want any more children
Age at last childbirth (years)	

Co-variates with categories	Description of categories of the co-variates
<20	Woman age was below 20 years at her last child birth
20-24	Woman was 20 to 24 years old at her last child birth
25-29	Woman was 25 to 29 years old at her last child birth
30+	Woman was 30 or above at her last child birth
Parity	
1-2	Last birth was first or second order birth
3+	Last birth was third or higher order birth
Years of schooling	
≤5	Never attended school or completed the maximum of five years of schooling
6-9	Completed 6-9 years of schooling
≥10	Completed 10 or more years of schooling
Household wealth quintiles (HWQ)	
Lower two HWQs	Member of a household that falls in the lower two wealth quintiles
Middle HWQ	Member of a household that falls in the middle two wealth quintiles
Upper two HWQs	Member of a household that falls in the upper two wealth quintiles
Religious affiliation	
Islam	Muslim
Other	Non-Muslim; non-Muslims in Bangladesh are predominantly Hindus
Geographical region	
West	Lives in Khulna, Rajshahi or Rangpur divisions
Central	Lives in Dhaka, Mymensingh or Barishal divisions
East	Lives in Chattogram or Sylhet divisions
Residence type	
Rural	Lives in a rural area
Urban	Lives in an urban area

Appendix S2. FP imitation within 12 months postpartum

Factors	Model I		Model II	
	AHR	95% CI	AHR	95% CI
PPFP counselling during ANC				
Didn't receive	Ref.	-	Ref.	-
Received	1.04	[0.94,1.16]	1.06	[0.89,1.26]
No ANC	1.09	[0.95,1.26]	1.53^{**}	[1.16,2.01]
PPFP counselling during PNC				
Didn't receive	Ref.	-	Ref.	-
Received	1.19^{*}	[1.03,1.38]	1.22^{*}	[1.03,1.45]
Had PNC, but counselling information unknown	0.78	[0.46,1.33]	0.79	[0.46,1.36]
No PNC	0.77	[0.45,1.31]	0.78	[0.45,1.34]
FP counselling during ANC and PNC (interaction)				
Counselling at ANC, counselling at PNC	-	-	0.94	[0.71,1.24]
Counselling at ANC, counselling at PNC unknown			1.00	[0.78,1.28]
Counselling at ANC, No PNC	-	-	1.05	[0.75,1.47]
No ANC, counselling at PNC	-	-	3.10	[0.30,32.16]
No ANC, counselling at PNC unknown			0.68^{*}	[0.49,0.95]
No ANC, no PNC	-	-	0.68	[0.45,1.04]
Menstruation resumption				
Did not return	Ref.	-	Ref.	-
Within 3 months of last birth	6.16^{**}	[5.15,7.36]	6.20^{**}	[5.17,7.43]
Within 4-7 months of last birth	3.47^{**}	[2.90,4.14]	3.50^{**}	[2.92,4.20]
Within 8-11 months of last birth	2.23^{**}	[1.83,2.71]	2.25^{**}	[1.84,2.74]

**Factors**	**Model I**		**Model II**	
	**AHR**	**95% CI**	**AHR**	**95% CI**
TLC vs. DNC				
TLC < DNC	Ref.	-	Ref.	-
TLC = DNC	1.22**	[1.11,1.33]	1.21**	[1.11,1.33]
TLC > DNC	1.27**	[1.08,1.50]	1.27**	[1.08,1.50]
Other	0.83	[0.41,1.69]	0.83	[0.41,1.70]
Place of birth				
Home	Ref.	-	Ref.	-
Facility	0.78	[0.46,1.32]	0.77	[0.45,1.31]
Mode of delivery				
Normal	Ref.	-	Ref.	-
C-section	0.99	[0.89,1.10]	0.99	[0.90,1.10]
Wantedness of last child				
Wanted then or later	Ref.	-	Ref.	-
Wanted no more	1.26**	[1.08,1.46]	1.26**	[1.08,1.47]
Age at last childbirth (years)				
<20	Ref.	-	Ref.	-
20-24	0.92	[0.84,1.01]	0.92	[0.83,1.01]
25-29	0.88*	[0.78,0.98]	0.88*	[0.78,0.98]
30+	0.86*	[0.75,0.99]	0.86*	[0.75,0.98]
Parity [ref.: 1-2]				
1-2	Ref.	-	Ref.	-
3+	1.00	[0.87,1.14]	1.00	[0.87,1.14]
Years of schooling				
<=5	Ref.	-	Ref.	-
6-9	0.95	[0.87,1.03]	0.95	[0.87,1.03]
10+	1.07	[0.96,1.19]	1.07	[0.96,1.19]
Household wealth quintiles				
I+II	Ref.	-	Ref.	-
III	0.93	[0.84,1.03]	0.93	[0.84,1.03]
IV+V	0.93	[0.84,1.03]	0.93	[0.85,1.03]
Religious affiliation				
Islam				
Other	0.97	[0.86,1.10]	0.98	[0.86,1.11]
Administrative division				
Chattogram	Ref.	-	Ref.	-
Barishal	1.10	[0.94,1.28]	1.09	[0.94,1.27]
Dhaka	1.17*	[1.01,1.36]	1.17*	[1.01,1.35]
Khulna	1.25**	[1.08,1.45]	1.25**	[1.07,1.45]
Mymensingh	1.26**	[1.09,1.46]	1.26**	[1.09,1.46]
Rajshahi	1.24**	[1.06,1.45]	1.24**	[1.06,1.45]
Rangpur	1.26**	[1.08,1.48]	1.26**	[1.08,1.48]
Sylhet	1.07	[0.90,1.27]	1.07	[0.90,1.27]
Residence type				
Rural	Ref.	-	Ref.	-
Urban	1.17**	[1.07,1.28]	1.17**	[1.07,1.29]
Observations	4784		4784	
